# Association of Albumin–Bilirubin (ALBI) Grade With 28-Day All-Cause Mortality in Patients With Acute Respiratory Distress Syndrome: A Retrospective Analysis of the MIMIC-IV Database

**DOI:** 10.1155/mi/9930648

**Published:** 2025-07-14

**Authors:** Weixiao Chen, Qingzhou Chen, Zhiwei Tang, Zhibo Li, Weiyan Chen, Xuming Xiong, Deliang Wen, Zhenhui Zhang

**Affiliations:** ^1^Department of Critical Care, The Second Affiliated Hospital of Guangzhou Medical University, No. 250 Changgang East Road, Haizhu District, Guangzhou, China; ^2^State Key Laboratory of Respiratory Disease, Clinical Laboratory Medicine Department, Guangzhou, China

**Keywords:** 28 days, acute respiratory distress syndrome, albumin-bilirubin, all-cause mortality, cohort, prognosis

## Abstract

The albumin–bilirubin (ALBI) grade, a validated prognostic tool in cancers such as hepatocellular carcinoma, has not been evaluated in acute respiratory distress syndrome (ARDS). This retrospective cohort study, utilizing data from the MIMIC-IV (v3.0) database, aimed to assess ALBI's predictive value for 28-day all-cause mortality in 338 adult ARDS patients admitted to the ICU. Patients were stratified into survivors (209 cases) and nonsurvivors (129 cases), with a 28-day mortality rate of 38.2%. Multivariable Cox regression identified ALBI as an independent predictor of 28-day mortality (HR = 1.46, 95% CI: 1.09–1.95, *p*=0.011). Receiver operating characteristic (ROC) analysis yielded an area under the curve (AUC) of 61.1% (95% CI: 54.7%–67.4%) with an optimal ALBI cutoff of −1.681; Kaplan–Meier (KM) survival curves confirmed significantly higher mortality in patients with ALBI ≥−1.681 versus ALBI <−1.681 (*p*=0.0098). Subgroup analyses revealed no significant interactions between ALBI and clinical variables (interaction *p*: 0.672–0.85). These findings demonstrate ALBI's utility as a novel, independent prognostic marker for short-term mortality risk in ARDS patients.

## 1. Introduction

Acute respiratory distress syndrome (ARDS) is a severe complication of respiratory diseases, with an annual incidence rate of approximately 75–90 cases per 100,000 people [1]. ARDS is a state of pulmonary inflammation, where damage to alveolar epithelial cells and the accumulation of fluid within the alveoli lead to localized oxygenation impairment [2, 3]. As the condition progresses, it may trigger systemic inflammatory response syndrome and even multiple organ dysfunction. The prognosis of ARDS is closely related to the severity of the disease, with about 60%–70% of patients having milder conditions that can gradually recover through supportive treatments, such as oxygen therapy and mechanical ventilation [4]. However, approximately 30%–40% of patients have severe conditions, characterized by severe hypoxemia and pulmonary insufficiency, with an overall mortality rate of 30%–50% [4, 5]. Therefore, timely assessment of the disease severity and effective treatment measures are key to reducing mortality. Current scoring systems for assessing the severity of ARDS include the Berlin definition, lung injury prediction score (LIPS) [6, 7], and Acute Physiology and Chronic Health Evaluation II (APACHE II) [8, 9]. These scoring systems typically require the integration of multiple clinical parameters, which may affect patient treatment outcomes due to assessment delays. Thus, developing a rapid, simple, and highly sensitive and specific biomarker to predict the severity and prognosis of ARDS is of great significance for improving patient survival rates [10, 11].

The albumin–bilirubin (ALBI) grade is an emerging tool for liver function assessment, predicting the prognosis of patients with liver diseases through serum albumin and bilirubin levels [12–14]. The ALBI score has been proven to reflect the synthetic function of the liver and the metabolic state of bilirubin, providing clinicians with a simple and effective assessment method. It has shown good accuracy and reliability in predicting the prognosis of patients with cirrhosis, hepatocellular carcinoma, and liver transplants [13, 15]. In physiological mechanisms, serum albumin is not only an important plasma protein but also has various biological functions, including maintaining plasma colloid osmotic pressure, transporting various substances, and participating in immune regulation [16–19]. ARDS patients often have systemic inflammatory responses, which may lead to changes in serum albumin levels, as albumin is a negative acute-phase reactant, and its levels are associated with the severity of inflammation, disease prognosis, and mortality. Additionally, bilirubin, as an important indicator of liver metabolism, its abnormal increase is usually associated with liver dysfunction, and changes in liver function in ARDS patients may affect bilirubin metabolism, thereby affecting the ALBI score [20, 21]. To gain a deeper understanding of the relationship between the ALBI score and ARDS, we assessed the effectiveness of the ALBI grade as a potential predictive factor for the severity and prognosis of ARDS. Although the exact link between ALBI and ARDS patient mortality is currently unclear, we analyzed ARDS patients admitted between 2008 and 2022 in the MIMIC-IV (v3.0) database. Through these studies, we hope to reveal the potential value of the ALBI score in the management of ARDS patients, providing clinicians with a more comprehensive assessment tool to predict disease severity and prognosis, thereby guiding treatment decisions and improving patient survival rates.

## 2. Methods

### 2.1. Database Introduction

The data for this study were obtained from the MIMIC-IV (v3.0) database [22], which is a large, publicly accessible database developed and managed by the Massachusetts Institute of Technology's Laboratory for Computational Physiology (https://physionet.org/content/mimiciv/3.0/). This database encompasses information on all patients admitted to the Beth Israel Deaconess Medical Center (BIDMC) from 2008 to 2022. It records each patient's hospital stay, laboratory tests, medication treatments, vital signs, and other comprehensive information. To protect patient privacy, all personal information has been de-identified, with random codes replacing patient identification, thus eliminating the need for patient consent and ethical approval. The MIMIC-IV (v3.0) database can be downloaded from the PhysioNet online forum (https://physionet.org/). To access the database, the first author of this study, Weixiao Chen, completed the Collaborative Institutional Training Initiative (CITI) Program and passed the “Conflict of Interest” and “Research Involving Only Data or Specimens” exams (ID: 10311970). The research team was ultimately qualified to use the database and extract data.

### 2.2. Population Selection Criteria

**Figure 1:**
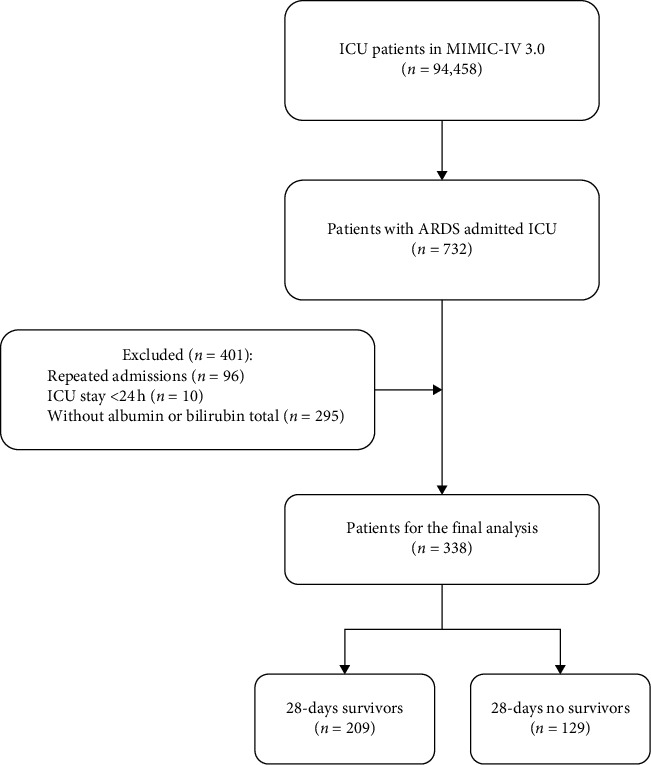
Schematic diagram of study sample selection steps. ALBI, albumin–bilirubin grade; ARDS, acute respiratory distress syndrome; ICU, intensive care unit; MIMIC, medical information mart for intensive care.

The MIMIC-IV (v3.0) database records a total of 546,028 hospital admissions, of which 94,458 were admitted to the ICU. Admission information for ARDS patients was extracted using the International Classification of Diseases, 9th revision (ICD-9) code 577.0 and the International Classification of Diseases, 10th revision (ICD-10) codes K85–K85.92, totaling 633 ICU-admitted patients. After further screening, patients meeting the following criteria were excluded: (1) patients under 18 years of age at the time of their first admission; (2) patients readmitted for ARDS, with only the first admission data retained; (3) patients with an ICU stay of less than 24 h; and (4) patients without blood lactate and serum albumin data recorded within 24 h of admission. Ultimately, this study included 338 patients (Figure 1).

### 2.3. Data Extraction

ALBI was selected as the primary study variable. Serum albumin and bilirubin concentrations were measured for the first time after admission to minimize the interference of subsequent treatments on albumin and bilirubin values. Potential confounding factors extracted included demographics (age, gender, and race); vital signs (heart rate, systolic blood pressure, diastolic blood pressure, mean arterial pressure, and respiratory rate); clinical treatments (use of vasopressin, octreotide, statins, beta-blockers, and metformin, mechanical ventilation, continuous renal replacement therapy, endoscopic retrograde cholangiopancreatography); comorbidities (acute kidney injury, sepsis, respiratory failure, heart failure, atrial fibrillation, hypertension, diabetes, and obesity); laboratory indicators (red blood cells, white blood cells, red cell distribution width (RDW), platelets, hemoglobin, lymphocyte percentage, hematocrit, total bilirubin, glutathione, serum glucose, serum creatinine, blood urea nitrogen, anion gap, prothrombin time, international normalized ratio, serum potassium, serum sodium, and serum calcium); Acute Physiological Score Ⅲ (APS Ⅲ); Systemic Inflammatory Response Syndrome Score (SIRS); and Sequential Organ Failure Assessment (SOFA). Data extraction tools used PostgreSQL software (v13.7.1) and Navicat Premium software (version 15) to extract data through running structured query language (SQL). All codes used for calculating demographic characteristics, laboratory indicators, comorbidities, and severity scores were obtained from the GitHub website (GitHub-MIT-LCP/mimic-iv: deprecated. For the latest MIMIC-IV (v3.0) codes, please refer to: https://github.com/MIT-LCP/mimic-code).

### 2.4. Grouping and Terminal Node Timing

In this study, patient subgroups were divided into a 28-day survival group after ICU admission (*n* = 209) and a 28-day mortality group after ICU admission (*n* = 129). The primary study endpoint was all-cause mortality within 28 days after ICU admission. The 28-day all-cause mortality rate is defined as the ratio of the total number of all-cause deaths during the 28-day ICU stay to the average number of people in the same population during the same period.

### 2.5. Management of Missing Data and Outliers

To avoid bias, variables with missing values exceeding 15% were excluded, such as HbA1c, lipoprotein profile, C-reactive protein, high-sensitivity CRP, aspartate aminotransferase, and patient height or weight. Variables with missing values greater than 5% and less than 15% (lymphocyte percentage, PT, INR) were processed using decision tree imputation to select the relatively optimal dataset to fill in the missing values. On the other hand, variables with missing values less than 5% (heart rate, systolic blood pressure, diastolic blood pressure, mean arterial pressure, respiratory rate, red blood cells, white blood cells, RDW, platelets, hemoglobin, hematocrit, glutathione transaminase, blood urea nitrogen, serum calcium) were replaced with the average value of the variable. Variables with outliers were processed using the winsorize method, using the winsor2 command, with 1% and 99% as cutoff points. Missing and outlier data were processed using STATA software (version 17).

### 2.6. Study Design

The discriminatory ability of the ALBI score was evaluated using receiver operating characteristic (ROC) curve analysis and the area under the curve (AUC). An AUC >0.5 indicated good discriminatory performance. LASSO regression was applied to reduce dimensionality among variables with potential correlations, selecting features with nonzero coefficients. We assessed multicollinearity among variables using the variance inflation factor (VIF), and variables with VIF values >5 (indicating significant multicollinearity) were excluded. Kaplan–Meier (KM) survival curves were employed to analyze survival outcomes between the two groups. Forest plots were used to visualize the presence or absence of significant interaction effects between ALBI and subgroup variables.

### 2.7. Statistical Analysis

The normality of continuous variables was assessed using the Kolmogorov–Smirnov test. Continuous variables are expressed as the mean ± SD for normally distributed variables, median (IQR) for non-normally distributed continuous variables, and number (%) for categorical variables. In analyzing baseline characteristics, continuous variables were compared using *t*-tests or one-way ANOVA, and categorical variables were compared using Pearson *χ*2 tests and Fisher tests. Lasso regression and VIF were used to screen for the risk factors. Potential risk factors were identified using univariate Cox regression analysis, and variables with *p*-values less than 0.1 were included in multivariate Cox regression analysis to determine independent risk factors for in-hospital mortality. The predictive ability of ALBI for ARDS mortality within 28 days of admission, as well as the sensitivity and specificity of various indicators, and the calculation of the AUC, were assessed using ROC analysis. The optimal cutoff value for ALBI was determined by the Youden index and used to divide ALBI into high and low value groups. Subsequently, unadjusted survival curves were plotted using the KM method, and the two groups were compared using the logRank test. Finally, subgroup analyses were also conducted to investigate whether ALBI had any effects on different subgroups, including age, gender, race, acute kidney injury, sepsis, obesity, hypertension, respiratory failure, diabetes, atrial fibrillation, heart failure, and serum sodium concentration. All analyses were performed using free statistical software version 1.6 and the statistical software package R 4.1.1. Two-tailed tests with *p* < 0.05 were considered statistically significant.

## 3. Results

### 3.1. Baseline Demographics and Clinical Characteristics

The baseline characteristics of the 28-day survival and nonsurvival groups admitted to the ICU are listed in Table 1. Among 338 eligible patients (135 females [39.9%], 203 males [60.1%]), the median age was 58.1 years (IQR 43–73.2), with an overall 28-day mortality rate of 38.2%. We observed that, compared to the 28-day survival group, patients who did not survive ARDS were older (*p* < 0.001), and had higher Charlson, SAPS II, and SOFA scores. Laboratory indicators showed that the nonsurvival group had higher ALBI upon admission compared to the survival group (−1.5 [−2.2, 0.8] vs. −1.8 [−2.3, −1.3], *p* < 0.001), along with significantly higher WBC, serum creatinine, BUN, total bilirubin, and lactate levels (*p* < 0.05), while RBC and platelet count were lower. No significant differences were observed in other covariates (*p* > 0.05).

### 3.2. ALBI as an Independent Risk Factor for All-Cause Mortality Within 28 Days of ICU Admission

Variables demonstrating significant differences (*p* < 0.05) in Table 1 were incorporated into univariate Cox regression analysis. As shown in Table 2, unadjusted ALBI exhibited a significant association with 28-day all-cause mortality (HR = 1.46, 95% CI: 1.11–1.92, *p*=0.007). Parameters were selected using Lasso regression, and the coefficient variation characteristics of these variables are shown in Figure S1A. The 10-fold cross-validation method was applied to iterative analysis, and a model with excellent performance and the minimal number of variables was obtained when *λ* reached 100 (log *λ* = 2) (Figure S1B). The screened variables included age, CRRT, hyperlipidemia, AKI_stage, PT, glucose, and SPO2. All these variables showed VIF values below 5 (Figure S1C), indicating no multicollinearity in our model. Subsequently, covariates and potential risk factors were selected based on parameters screened by Lasso regression and included in the multivariate Cox regression analysis.  Table 3 presents the adjusted analysis of ALBI and all-cause mortality within 28 days of ICU admission for ARDS patients using the Cox proportional hazards model. In Model I, ALBI was associated with all-cause mortality within 28 days of ICU admission (HR, 1.46; 95% CI: 1.09–1.95; *p*=0.011), adjusting for confounding factors in the Cox regression table. This suggests that ALBI is an independent predictive factor for ARDS.

### 3.3. ROC Curve Analysis and KM Curve

**Figure 2:**
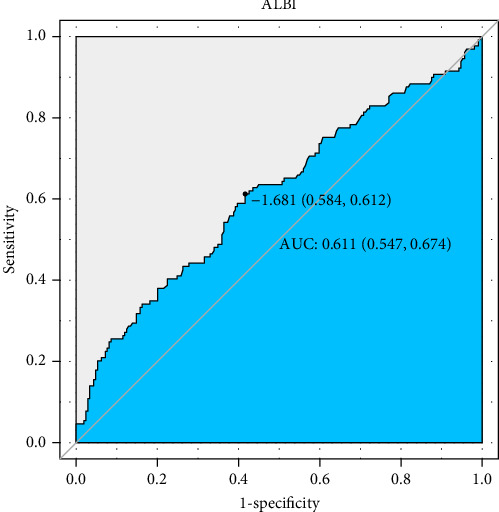
ROC curves of peripheral differential albumin–bilirubin correlate for predicting in-hospital mortality. The black solid line indicates the ROC curve for albumin–bilirubin. ALBI, albumin–bilirubin.

**Figure 3:**
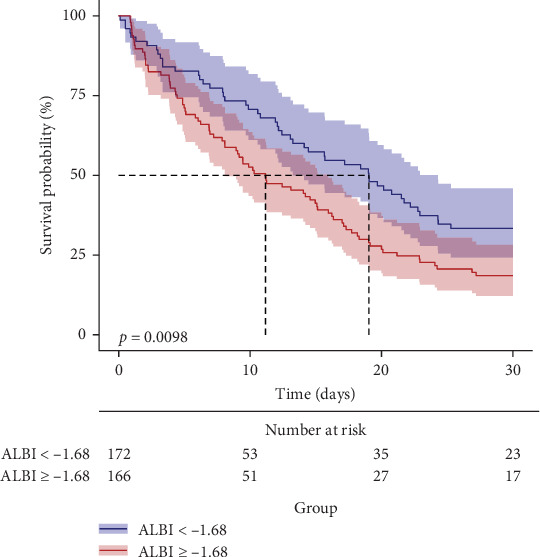
Kaplan–Meier survival analysis curves for all-cause mortality within 28 days of hospital admission.

The predictive performance of ALBI for 28-day all-cause mortality in ARDS patients was evaluated through ROC curve analysis (Figure 2, Table 4). The AUC for ALBI was (61.1% [95% CI: 54.7%–67.4%]), indicating that ALBI has good predictive power. At the same time, we obtained the optimal cutoff value of −1.681 for ALBI, with a sensitivity of 58.4% and a specificity of 61.2% (Figure 2). Using this threshold, patients were stratified into high ALBI (≥−1.681, *n* = 166) and low ALBI (<−1.681, *n* = 172) cohorts. KM survival analysis curves were plotted (Figure 3), and the results showed that the mortality rate of patients in the high-value group was significantly higher than that of the low-value group (*p*=0.0098).

### 3.4. Subgroup Analysis

**Figure 4:**
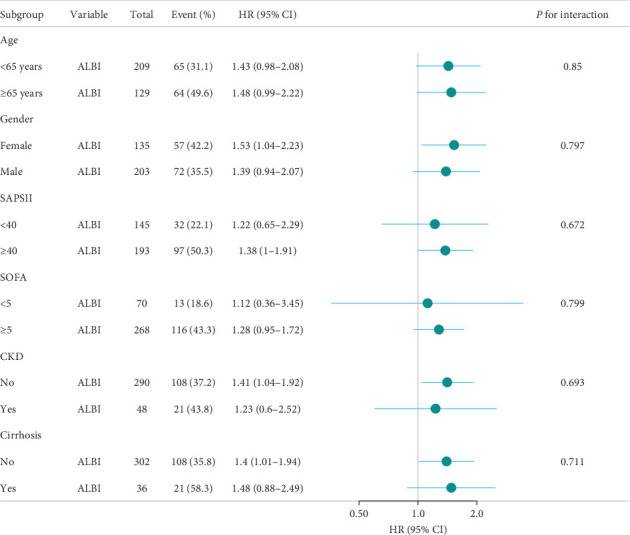
Forest plot for subgroup analysis of the relationship between hospital mortality and ALBI. ALBI, albumin–bilirubin; CKD, chronic kidney disease; SAPSⅡ, simplified acute physiology score Ⅱ; SOFA, sequential organ failure assessment.

Figure 4 assesses the stability of ALBI's association with 28-day mortality in ARDS patients' admission to the ICU across clinically relevant subgroups. When stratified analysis was conducted for age, gender, SAPSⅡ, SOFA, CKD and cirrhosis, the forest plot (Figure 4) showed no significant interaction between ALBI and each subgroup (interaction *p*: 0.672–0.85). These findings confirm ALBI's robustness as an independent predictor of short-term mortality in ARDS patients.

## 4. Discussion

This retrospective cohort study establishes the ALBI score as an independent predictor of 28-day all-cause mortality in patients with ARDS. The ROC curve analysis revealed that ALBI exhibited moderate predictive accuracy, with an AUC of 61.1% (95% CI: 54.7%–67.4%). Additionally, KM survival analysis demonstrates that ARDS patients with an ALBI score ≥−1.681 have significantly higher all-cause mortality within 28 days of admission compared to those with an ALBI score <−1.681, further supporting our findings.

Emerging research focuses on identifying novel biomarkers to predict ARDS prognosis. Although studies have investigated the role of systemic inflammatory biomarkers, such as RDW [23], neutrophil-to-lymphocyte ratio (NLR) [24, 25], RDW-to-platelet ratio (RPR) [26], glucose-to-lymphocyte ratio (GLR) [27], and C-reactive protein-to-albumin ratio (CRP/Alb) [28] in predicting ARDS prognosis, the ALBI score uniquely integrates hepatic and inflammatory profiles, offering a multidimensional assessment tool for ARDS prognosis. Although validated in heart failure and sepsis mortality risk stratification [29], ALBI's utility in ARDS remains underexplored, highlighting a critical gap in critical care prognostication. As a scoring system that reflects liver function and inflammatory status, the ALBI score has shown potential in predicting the prognosis of ARDS patients. By combining serum albumin and bilirubin levels, the ALBI score provides clinicians with a powerful tool to assess patients' liver function and inflammatory responses [13]. In the pathological process of ARDS, the metabolic function of the liver and inflammatory responses play a significant role in disease progression and prognosis.

Our findings confirm the ALBI score's association with adverse outcomes in critical illness, aligning with prior evidence of its prognostic value in sepsis [30]. However, the ALBI score may be influenced by various factors; patients with liver disease may have abnormal bilirubin metabolism, which could affect the accuracy of the ALBI score. In addition, certain medications, such as some antibiotics and chemotherapeutic drugs, can also cause changes in serum bilirubin levels. This requires us to base the ALBI score on the first measurement of serum albumin and bilirubin concentration after admission to minimize the interference of subsequent treatments on albumin and bilirubin values. Furthermore, some ARDS patients may show normal albumin levels in venous blood, which could reduce the reliability of the ALBI score in predicting patient prognosis. In these cases, the ALBI score may need to be combined with other biomarkers or clinical scoring systems to improve the predictive accuracy of ARDS patient prognosis. For example, combining the ALBI score with the Acute Physiology and Chronic Health Evaluation II (APACHE-II) score or the SOFA score may provide clinicians with a more comprehensive prognosis assessment tool.

ARDS is a group of acute diffuse pulmonary inflammatory response syndromes, characterized by respiratory distress and refractory hypoxemia [2, 3]. Pneumonia is the most common cause of ARDS. Due to its severe systemic inflammatory response, associated with diffuse alveolar damage leading to refractory hypoxemia, and prone to multiple organ failure, ARDS has become a challenging critical illness in the ICU [1, 31]. To some extent, the clinical presentation of ARDS is similar to that of patients with septic shock. In our study, by comparing the optimal critical values, we found that the best critical value for predicting all-cause mortality within 28 days of admission for ARDS patients was −1.681, while Su et al.'s [29] study found the best critical value for the ALBI score to be −2.32. This may be due to the different underlying diseases in our study. Therefore, the optimal critical values for different admission causes still need to be determined through prospective studies. Notably, Gou et al.'s [30] large-scale analysis (including 11,810 patients) corroborates ALBI's prognostic accuracy in sepsis-related critical illness, aligning with our findings. Therefore, integrating ALBI monitoring into clinical workflows could enhance risk stratification and guide personalized management of ARDS patients.

Our study has several limitations. First, our study is a single-center retrospective cohort study, which cannot clarify the relationship between ALBI and ARDS like a prospective study, making our results less convincing. Second, we studied the relationship between the ALBI measured for the first time after admission and prognosis, which does not allow us to dynamically assess the impact of ALBI on prognosis. Finally, the population data we studied came from MIMIC-IV (v3.0), which covers hospitalized patients from 2008 to 2019. With the development of drug treatment and optimization of treatment plans, such a long period cannot guarantee the consistency of treatment plans for patients admitted around that time, and we cannot determine whether this will lead to bias in the study results.

## 5. Conclusion

In summary, the ALBI score can be used as an independent predictor of all-cause mortality within 28 days of admission for ARDS patients in our study, providing healthcare workers with better tools for timely early intervention and clinical planning for adverse patient outcomes. As an objective biomarker readily available, LAR still needs further validation in large-scale, multicenter, prospective studies.

## Figures and Tables

**Table 1:** Baseline characteristics between survivors and nonsurvivors.

Variables	Total (*n* = 338)	28-days survivor (*n* = 209)	28-days no survivor (*n* = 129)	*p*-Value
ALBI, mean ± SD	−1.7 ± 0.6	−1.8 ± 0.5	−1.5 ± 0.7	<0.001
Age (years), mean ± SD	58.1 ± 15.1	56.0 ± 15.5	61.6 ± 13.6	<0.001
Gender (male), *n* (%)	203 (60.1)	131 (62.7)	72 (55.8)	0.211
WBC (K/µL), median (IQR)	12.9 (9.0, 17.9)	11.7 (8.2, 17.7)	13.9 (9.9, 18.6)	0.048
RBC (K/µL), mean ± SD	3.7 ± 0.9	3.8 ± 0.9	3.6 ± 0.9	0.041
Platelet count (K/µL), mean ± SD	218.9 ± 118.3	234.5 ± 110.9	193.6 ± 125.8	0.002
Bilirubin total (µmol/L), mean ± SD	38.0 ± 90.0	25.7 ± 69.0	57.8 ± 113.7	0.001
Albumin (g/L), mean ± SD	28.7 ± 6.1	29.1 ± 5.7	27.9 ± 6.5	0.061
pH, mean ± SD	7.3 ± 0.1	7.3 ± 0.1	7.3 ± 0.1	0.001
PCO_2_ (mmHg), mean ± SD	45.7 ± 11.3	44.8 ± 10.6	47.1 ± 12.1	0.069
PO_2_ (mmHg), mean ± SD	96.8 ± 34.7	98.2 ± 35.3	94.5 ± 33.6	0.334
Lactate (mmol/L), mean ± SD	2.6 ± 2.4	2.2 ± 1.9	3.2 ± 3.0	<0.001
Urea nitrogen (mg/dL), median (IQR)	26.0 (16.5, 42.6)	22.0 (14.5, 35.7)	34.3 (21.0, 49.5)	<0.001
Creatinine (mg/dL), median (IQR)	1.2 (0.8, 2.1)	1.1 (0.8, 1.8)	1.6 (1.0, 2.8)	<0.001
SOFA, mean ± SD	8.0 (5.0, 11.0)	7.0 (4.0, 10.0)	10.0 (6.0, 13.0)	<0.001
SapsII, mean ± SD	44.2 ± 16.3	39.8 ± 14.0	51.3 ± 17.2	<0.001
Charlson, mean ± SD	3.8 ± 2.8	3.4 ± 2.9	4.4 ± 2.4	0.001
Hypertension, *n* (%)	115 (34.0)	72 (34.4)	43 (33.3)	0.833
Diabetes, *n* (%)	90 (26.6)	56 (26.8)	34 (26.4)	0.930
Heart failure, *n* (%)	60 (17.8)	32 (15.3)	28 (21.7)	0.135
Malignant tumor, *n* (%)	16 (4.7)	10 (4.8)	6 (4.7)	0.955
Chronic kidney disease, *n* (%)	48 (14.2)	27 (12.9)	21 (16.3)	0.390
Cirrhosis, *n* (%)	36 (10.7)	15 (7.2)	21 (16.3)	0.008
Chronic obstructive pulmonary disease, *n* (%)	34 (10.1)	18 (8.6)	16 (12.4)	0.260
Hyperlipidemia, *n* (%)	103 (30.5)	54 (25.8)	49 (38)	0.018
Acute kidney injury, *n* (%)	326 (96.4)	199 (95.2)	127 (98.4)	0.141
Acute kidney injury stage, *n* (%)				<0.001
1	30 (8.9)	23 (11)	7 (5.4)	
2	116 (34.3)	90 (43.1)	26 (20.2)	
3	180 (53.3)	86 (41.1)	94 (72.9)	
Continuous renal replacement therapy, *n* (%)	111 (32.8)	43 (20.6)	68 (52.7)	<0.001
Ventilation, *n* (%)	284 (84.0)	173 (82.8)	111 (86)	0.425
Ventilation time (h), median (IQR)	171.6 (73.7, 332.9)	200.5 (105.0, 382.7)	133.8 (54.9, 259.9)	<0.001

Abbreviations: ALBI, albumin–bilirubin; RBC, red blood cell; SAPSⅡ, simplified acute physiology score Ⅱ; SOFA, sequential organ failure assessment; WBC, white blood cell.

**Table 2:** Univariate Cox analysis of risk factors for death within 28 days in patients by logistic regression analysis.

Variables	HR (95% CI)	*p*-Value (Wald's test)
ALBI (cont. var.)	1.46 (1.11–1.92)	0.007
Age (cont. var.)	0.99 (0.97–1.00)	0.042
Gender: M vs. F	1.00(0.71–1.42)	0.973
Bilirubin total (cont. var.)	1.00 (1.00–1.00)	0.001
Albumin (cont. var.)	0.98 (0.95–1.01)	0.153
pH (cont. var.)	0.07 (0.01–0.44)	0.005
Lactate (cont. var.)	1.23 (1.15–1.32)	<0.001
Diabetes: Yes vs. no	0.85 (0.57–1.25)	0.4
Malignant tumor: Yes vs. no	0.68 (0.30–1.54)	0.353
Chronic kidney disease: Yes vs. no	0.60(0.37–0.96)	0.032
Cirrhosis: Yes vs. no	1.23 (0.77–1.96)	0.394
AKI: Yes vs. no	1.76 (0.43–7.12)	0.428
CRRT: Yes vs. no	1.4 (0.99–1.98)	0.057
Ventilation: Yes vs. no	1.4 (0.85–2.31)	0.185
Ventilation hour (cont. var.)	0.99 (0.99–0.99)	<0.001
SOFA (cont. var.)	1.09 (1.04–1.13)	<0.001
SAPSII (cont. var.)	1.02 (1.01–1.03)	<0.001
Charlson (cont. var.)	0.9 (0.84–0.96)	0.003

Abbreviations: AKI, acute kidney injury; ALBI, albumin–bilirubin; CRRT, continuous renal replacement therapy; SAPSⅡ, simplified acute physiology score Ⅱ; SOFA, sequential organ failure assessment.

**Table 3:** Multivariate Cox analysis of risk factors for death in patients within 28 days by logistic regression analysis.

Outcomes	n.event (%)	Univariable analysis		Multivariable analysis	
		HR (95% CI)	*p*-Value	HR (95% CI)	*p*-Value
Primary outcome					
28-day all-cause mortality	129 (38.2)	1.50 (1.14–1.98)	0.004	1.46 (1.09–1.95)	0.011
Secondary outcomes					
ICU mortality	126 (37.3)	1.53 (1.14–2.03)	0.004	1.38 (1.02–1.87)	0.036
In-hospital mortality	145 (42.9)	1.48(1.13–1.93)	0.005	1.40 (1.06–1.86)	0.019

**Table 4:** Information of ROC curves in Figure 2.

Variables	AUC (%)	95% CI (%)	Threshold	Sensitivity	Specificity
ALBI	61.08	54.74–67.41	−1.6807	0.6124	0.5837
Albumin	43.65	37.25–50.05	3.75	0.0775	0.9474
Bilirubin total	57.95	51.56–64.34	1.425	0.3256	0.8325

Abbreviation: ALBI, albumin–bilirubin.

## Data Availability

The datasets presented in this study can be found in online repositories. The names of the repository/repositories and accession numbers can be found in the article/supporting information.

## Supporting Information

Supporting Information Figure S1: Collinearity, high collinearity (VIF) may inflate parameter uncertainty. (A) Coefficients. (B) Binomial deviance. (C) Variance inflation factor.
