# Supplementary figures and images for: Association of Albumin–Bilirubin (ALBI) Grade With 28-Day All-Cause Mortality in Patients With Acute Respiratory Distress Syndrome: A Retrospective Analysis of the MIMIC-IV Database
Source: Mediators Inflamm. 2025 Jul 14;2025:9930648. doi: 10.1155/mi/9930648 (PMC12279418; doi:10.1155/mi/9930648)

**A**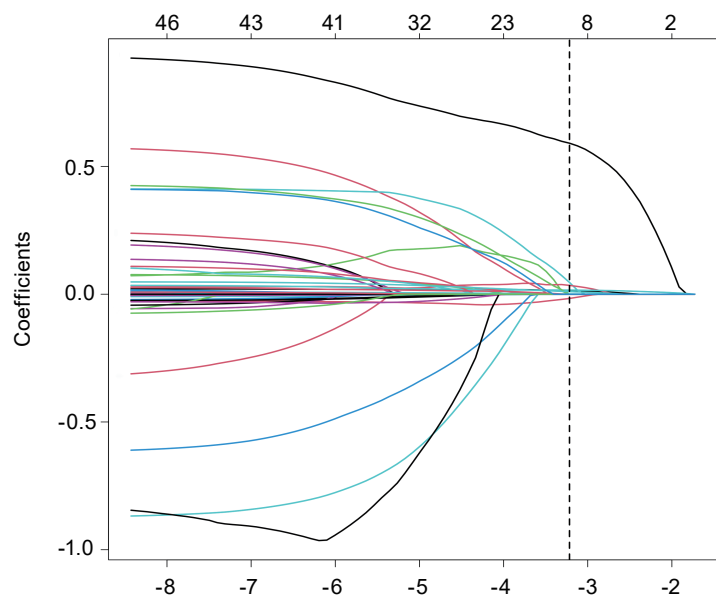**B**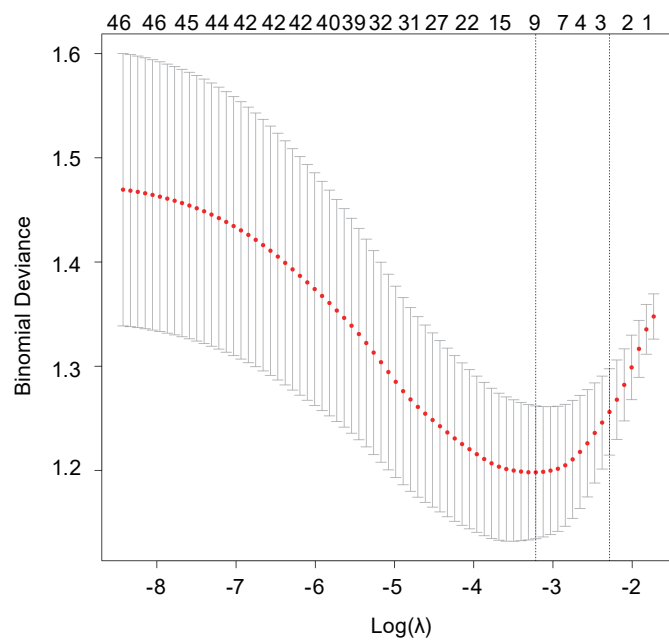**C**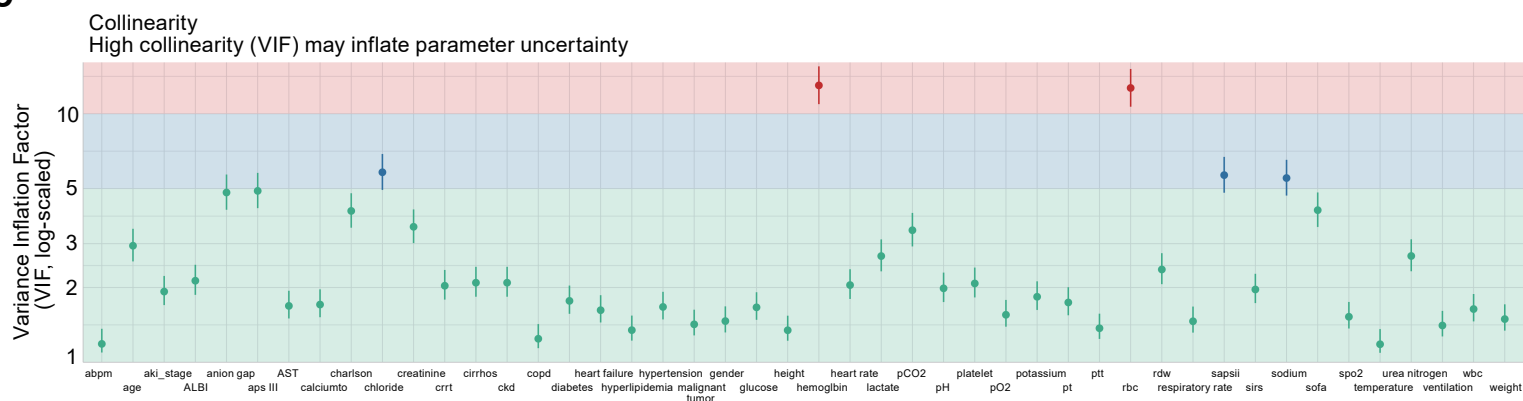

Supplement: Supporting Information — Figure S1: Collinearity, high collinearity (VIF) may inflate parameter uncertainty. (A) Coefficients. (B) Binomial deviance. (C) Variance inflation factor. [file 9930648.f1.pdf]
